# Anti-Inflammatory Properties of the Enaminone E121 in the Dextran Sulfate Sodium (DSS) Colitis Model

**DOI:** 10.1371/journal.pone.0168567

**Published:** 2016-12-20

**Authors:** Maitham A. Khajah, Kethireddy V. Ananthalakshmi, Ivan Edafiogho

**Affiliations:** 1 Faculty of Pharmacy, Kuwait University, Safat, Kuwait; 2 Department of Pharmaceutical Sciences, University of Saint Joseph School of Pharmacy, Hartford, Connecticut, United States of America; Future University, EGYPT

## Abstract

**Background:**

Enaminones are synthetic compounds with an established role in the prevention of various forms of seizures. Recent evidence suggests potent anti-tussive, bronchodilation and anti-inflammatory properties. Pre-treatment with particularly E121 compound resulted in a decrease in leukocyte recruitment in the ovalbumin induced-model of asthma, immune cell proliferation and cytokine release *in vitro*. We hypothesize that E121 might serve as a therapeutic potential in intestinal inflammation through modulating immune cell functions.

**Methods:**

Colitis was induced by daily dextran sulfate sodium (DSS) administration for 5 days, and its severity was determined by gross and histological assessments. The plasma level of various cytokines was measured using flow cytometry-based assay. The colonic expression/ phosphorylation level of various molecules was determined by immunofluorescence and western blotting. The effects of E121 treatment on *in vitro* neutrophil chemotaxis (under-agarose assay), superoxide release (luminol oxidation assay) and apoptosis (annexin V/7AAD) were also determined.

**Results:**

DSS-induced colitis in mice was significantly reduced by daily E121 treatment (30–100 mg/kg) at gross and histological levels. This effect was due to modulated plasma levels of interleukin (IL-2) and colonic expression levels of various signaling molecules and proteins involved in apoptosis. *In vitro* neutrophil survival, chemotaxis, and superoxide release were also reduced by E121 treatment.

**Conclusion:**

Our results indicate important anti-inflammatory actions of E121 in the pathogenesis of IBD.

## Introduction

Inflammatory bowel disease (IBD) is defined as a group of disorders affecting the gastrointestinal tract (GIT), which include two major conditions; Crohn’s disease (CD) and ulcerative colitis (UC) [1–5]. These two conditions share some pathological and clinical features but also have distinct factors involved in their pathogenesis. CD can affect any part of the GIT, and the inflammation occurs in a patchy pattern and can extend to the full thickness of the gut. In contrast, the inflammation in UC is limited to the mucosal and sub-mucosal layers of the rectum and colon, with a continuous pattern of distribution. Multiple factors play a role in the pathogenesis of IBD including environmental, genetic, immune and barrier defect. IBD is thought to result mainly from a dysregulated immune response to commensal bacteria in the gut in a genetically susceptible individual, whereas the environmental factors and barrier defects play a role in disease exacerbation [4, 6, 7]. Various immune cells contribute to the pathogenesis of IBD including T-cells, monocytes, and neutrophils. The immune response in CD is mainly driven through a T-helper (Th1) mediated response, as demonstrated by elevated expression of interleukins (IL-1β, -2, and -12), tumor necrosis factor (TNF-α), and interferon gamma (IFN-γ) [8–10]. In contrast, the immune response in UC is a Th2-mediated response with elevated levels of IL-5 and IL-13 (from NKT-cells) [11]. Evidence also shows an important role of the Th-17 in IBD pathogenesis. IL-23/IL-17 has been shown to be elevated in animal models of IBD, as well as in the serum and colonic tissues from CD and UC patients. In fact, several studies suggest an important pro-inflammatory role of Th-17 contributing to IBD pathogenesis [12–14]. In addition, the levels of the anti-inflammatory cytokines such as IL-10 and transforming growth factor-β (TGFβ) are decreased in both conditions relative to healthy individuals. Evidence also suggest the importance of reactive oxygen [15] and nitrogen species [16] in IBD pathogenesis, which contribute to chronic inflammation and tissue damage. For example, nitric oxide (NO) stimulates TNF-α production in the colon, leading to neutrophils recruitment to the gut in part through stimulating the synthesis of intracellular adhesion molecule (ICAM) and P-selectin [17]. In addition, enhanced expression/activity of the transcription factor NF-ƙB was noted in the colonic tissues and also in newly recruited immune cells to the gut in IBD patients. NF-ƙB activity in the colon lead to enhanced secretion of various pro-inflammatory cytokines such as IL-1, IL -6, IL -12, IL -23 and TNF-α [18–21], matrix metalloproteinases [22, 23], as well as enhanced expression of intercellular adhesion molecule-1(ICAM-1) [24].

Dextran sulfate sodium (DSS) induced colitis is one of the most widely used model in IBD research, due to its low cost, easy way of administration, as well as reproducibility of the inflammatory reaction. DSS produce clinical and histopathological features similar to those seen in human IBD [25]. The DSS is a water-soluble negatively charged sulfated polysaccharide with a wide range of molecular weight (5–1400 kDa) [26]. It is suggested that DSS passes through mucosal epithelial cells and binds with medium-chain-length fatty acids (MCFAs) in the colon forming nano-lipo complexes [27], and induces erosions in the colonic epithelial cells leading to impaired integrity and increased permeability. Moreover, the intestinal bleeding is caused by the anticoagulant property of DSS [26]. In addition, DSS modifies the expression level of tight junction proteins and up-regulates the expression of various pro-inflammatory cytokines, which is also seen in IBD patients [28]. Several symptoms are seen after DSS administration including weight loss, diarrhea, sticky bloody stools, increased colon thickness, and reduced colon length. Furthermore, histological changes in the colon include destruction of normal mucosal architecture, immune cell infiltration (e.g. neutrophils, macrophages, and T-cells influx into the lamina propria and submucosa), goblet cell depletion, and crypt abscesses [26, 29, 30].

Enaminones are synthetic compounds consisting of an amino group, joined through an alkene group to a ketone group [31–33]. They are known to possess various therapeutic effects as anti-convulsants and anti-malarial agents as well as having cardiovascular benefits [34, 35]. Their anti-convulsant effects are mediated in part through interaction with glutamate and its receptors [36], enhancing extracellular GABA levels (possibly through the inhibition of either GABA-T enzymatic activity, GABA re-uptake, or both mechanisms), and inhibiting tetrodotoxin-sensitive sodium currents [37, 38]. The enaminone E139 was shown to inhibit seizure activity *in vitro* which was induced both chemically and electrically [39]. A recent study showed that the modification of 2,4-dihalogenation of the phenylamino moiety of the enaminones results in more potent and efficacious anti-convulsant agents [40]. Recent evidence also suggests anti-inflammatory properties of the enaminones, particularly for the E121 compound [ethyl 4-(4`-chlorophenyl) amino-6-methyl-2-oxocyclohex-3-en-1-oate]. E121 (at doses of 100–200 mg/kg, given i.p) demonstrated a potent anti-tussive and bronchodilator effect in the citric acid-induced cough model in guinea pigs, through enhancing K_ATP_ channels and β2-receptor activity [41]. Furthermore, in a murine model of ovalbumin-induced asthma, E121 (at doses of 60–100 mg/kg, given i.p) significantly decreased broncho-alveolar lavage fluid (BALF) total leukocytes count and the percentage of eosinophils, and increased the relative number of macrophages. It also inhibited the proliferation of the peripheral blood human mononuclear cells and cytokine release particularly for IL-2, -4, -5, and TNFα *in vitro* (at a dose of 50μg/ml). These data suggest a potent anti-inflammatory effect of the E121 compound in part through modulating leukocyte recruitment and cytokine release from monocytes and T-cells such as TNFα, which all play a role in the pathogenesis of IBD as well.

In this study, we showed for the first time a potent anti-inflammatory property of E121 in experimental colitis using the DSS model. Daily intra-peritoneal (i.p) administration of E121 (at doses of 30–100 mg/kg) significantly reduced colitis severity at gross and histological levels. This effect was in part mediated through modulating the plasma levels of IL-2 and the expression/activity of various signaling molecules and proteins involved in cell apoptosis. Furthermore, E121 significantly enhanced spontaneous neutrophil apoptosis, and reduced fMLP-like peptide (WKYMVm)-induced neutrophil chemotaxis and superoxide release *in vitro*.

## Materials and Methods

The methods were carried out in accordance with Kuwait University roles and regulations.

### Synthesis of E121

The enaminone E121 was synthesized and characterized by Dr. Ivan Edafiogho by method previously reported [34, 36, 42–45].

### Animals

BALB/c mice (6–10 weeks old, mean weight 20 g.) were supplied by the Animal Resource Center of the Health Sciences Center at Kuwait University. All animals were kept under standard conditions including controlled temperature (25°C), a 12-h light-dark cycle and had free access to food and drinking water *ad libitum*. All experimentations were approved by the Animal Care Committee at Kuwait University Health Sciences Center and conformed to their rules and regulations as described previously [29].

### Induction of colitis

Colitis was induced in mice by delivering DSS polymers (3.5% w/v, m.wt 40 kD; MP Biomedicals, France) in autoclaved drinking water and provided *ad libitum* for 5 days as previously described [29, 46]. The following groups of mice (6 per group) were used: a) control (untreated; UT) mice received autoclaved tap water only. DSS was administered to b) UT mice, c) daily i.p vehicle treated mice, or daily i.p E121 treated mice at d) 10 mg/kg, e) 30 mg/kg, f) 60 mg/kg. and g) 100 mg/kg dose. E121 (purity 99.7%) was dissolved in 50% PEG, 30% PBS and 20% ethanol (vehicle), and freshly prepared from the stock each day of the experiment, and administered to mice by daily intra-peritoneal (i.p) injections in a volume of 500 μl per injection along with DSS treatment.

### Gross (macroscopic) assessment of colitis severity

The gross assessment for colitis severity was performed as described previously [29, 46].

The data are presented as the percentage (%) of mice in each group showing various features of colitis (Table 1).

**Table 1:** Effect of E121 treatment on the macroscopic score for colitis severity.

% of mice displaying each parameter					
Mice group	edema	diarrhea	erythema	blood in stool	anorectal bleeding
UT	0	0	0	0	0
DSS alone	90	90	90	80	80
DSS + Vehicle	100	100	100	80	80
DSS + E121 10mg/kg	100	70	100	50	75
DSS + E121 30mg/kg	100	20*	50*	20*	20*
DSS + E121 60mg/kg	100	0*	50*	0*	0*
DSS + E121 100mg/kg	100	0*	50*	0*	0*

Colitis severity (reflected by indicated parameters) was assessed in untreated (UT) mice or in mice receiving no treatment or daily i.p vehicle or E121 for 5 days along with DSS treatment. Numbers are the means of 6 mice for each group.

* denote significant difference from DSS alone and DSS/i.p vehicle treated mice.

### Histological assessment of colitis severity

Formalin fixed colons were processed for histological assessment, and were (blindly) scored by 2 observers using a standard semi-quantitative histology scoring system as previously described [29, 30, 46, 47].

### Differential white blood cell counts

Differential white blood cell (WBC) count was determined as previously described [29]. A total of 100 cells were randomly counted in each slide and the data are presented as % of the presence of each cell type [29].

### Western blotting

Colon tissue samples (descending part) were cut and homogenized (with Teflon glass homogenizer) in 1 ml of buffer composed of 1.2 g of 50 mM HEPES, 0.3 g of 50 mM NaCl, 1 ml of 0.5 M EDTA, 1 ml of 1% Triton X-100 and 98 ml of deionized water. A protease inhibitor cocktail (10 μg/ml aprotinin, 10 μg/ml leupeptin and 100 μM PMSF) was added separately. Homogenates were centrifuged at 1,800 rpm for 10 min at 4°C, and the supernatant collected. Protein concentration was determined by the Bradford assay (Bio-Rad, Hercules, CA). Samples containing 50 μg protein were dissolved in an equal volume of 2 x Lammeli sample buffer and β-mercaptoethanol, heated at 90°C for 10 min and loaded onto a 12.5% SDS-polyacrylamide gel and electrophoresed at 125 V for 1 h. Proteins were transferred (at 100 V for 1 h) onto a PVDF membrane (Millipore, Ireland) and then blocked with 4% BSA for 90 min before overnight incubation at 4°C with primary antibodies for actin (1/1000 dilution), PTEN, p-AKT, T-AKT, caspase 3 and 8 (1:500 dilution), and TRAF-2, BAX, RAF-1, SMAC, BCL-2, BCL-XL, and cytochrome c (1:200 dilution) (all from Cell Signaling Technology, Boston, MA, USA). Membranes were washed 3 times for 1 h with 1x TBS-T buffer and incubated with appropriate horseradish peroxidase (HRP)-labeled secondary antibodies [Anti-rabbit or Anti-mouse IgG, HRP linked antibody (Cell Signaling Technology, Boston, MA, USA; 1/1000 dilution)]. Bands were visualized using Super Signal ECL substrate (Thermo Scientific, Rockford, USA) and Kodak X-ray film.

### Immunofluorescence

Colon sections (5μm) were deparaffinized, rehydrated through a series of washes in graded ethanol and water, followed by an antigen retrieval step (by boiling the sections in 10 mM sodium citrate buffer, pH 6.0 for 20 min). Sections were then incubated in blocking solution (5% bovine serum albumin (BSA) + 0.3% Triton X-100 in PBS) for 1 h, followed by incubation overnight at 4°C with primary antibodies [p-ERK1/2, p-Akt, and p-p38 MAPK (1:100 dilution), Cell Signaling, USA, and Gr1 antibody (1:50 dilution), Thermo Scientific, USA] diluted in 1% blocking solution. On the following day, sections were washed and incubated with secondary antibody conjugated to Alexa Fluor 555 [Goat anti rabbit SFX kit; Life Technologies,USA (1:400 dilution) for 2 h at room temperature] in the dark. After washes in PBS sections were stained with 4’,6 diamidino-2- phenylindole and mounted. Images were captured on a ZIESS LSM 700 confocal microscope and fluorescence intensity estimated in defined fields using Image J software package.

### Measurement of plasma cytokine levels

The circulating level of various cytokines were measured using the mouse Th1/Th2/Th17 flow cytometry multi-plex panel kit (Cat# MMX171T, Antigenix, USA) according to the manufacture instructions. In brief, 15 μl of plasma taken from UT mice or mice treated with DSS for 5 days plus daily i.p injections of vehicle or E121 (60 mg/kg) were added to separate wells containing 45 μl of capture bead working suspension. The plate was incubated on a plate shaker (700 rpm, 30 min in dark), the wells were then washed 3 times with washing buffer, and the solution was decanted. Then, 25 μl of biotinylated antibody working solution was added to each well, incubated on a plate shaker (700 rpm, 30 min in dark), washed 3 times using washing buffer, and the solution was decanted. After that, 25 μl of streptavidin-PE solution was added to each well, incubated on a plate shaker (700 rpm, 20 min in dark), and the solution was decanted. The wells were then washed twice with washing buffer, and the solution was decanted. This was followed by the addition of 150 μl of reading buffer to each well to re-suspend the beads and read using the flow cytometer (Beckman FC 500) at 488 nm excitation, and 700 nm emission.

### Isolation of bone-marrow derived neutrophils

Neutrophils were isolated from mouse bone marrow as previously described [48–50].

### Apoptosis assay

Freshly isolated bone-marrow derived neutrophils were re-suspended (10^6^ cells/ml) in RPMI containing 20% FBS and various doses of E121 (10 ng/ml– 100 μg/ml) or vehicle, plated in 35 mm culture dishes and incubated for 16 h in a 37°C/ 5% CO_2_ incubator. Subsequently, cells were washed twice (by re-suspension and low speed centrifugation) with ice-cold PBS and once with 1x Annexin-V binding buffer [10 mM HEPES/NaOH (pH 7.4), 140 mM NaCl, 2.5 mM CaCl_2_]. Pelleted cells were re-suspended in Annexin-V binding buffer and stained for FACS analysis using the PE Annexin V apoptosis detection kit I from BD Pharmingen as per the manufacturer’s protocol.

### Under-agarose chemotaxis assay

The under-agarose assay was performed as described previously [48, 50–52]. Three wells (3.5 mm) were created in a layer of sterile 0.5% agarose (2.5 mm apart) in 35 mm culture dishes. The center well was loaded with 10 μl of 1 μM fMLP-like peptide WKYMVm (Phoenix Pharmaceuticals, USA) as chemoattractant (or PBS for control condition) and the outer wells with 10 μl (10^7^ cells/ml) neutrophils [either vehicle treated, or pre-treated with various doses of E121 (10 ng/ml–10 μg/ml) for 1 h] and left for 4 h in a 37°C/5% CO_2_ incubator. Chemotaxis was visualized microscopically and determined by subtracting the number of neutrophils which migrated toward the chemoattractant (or PBS) well from the number migrating in the opposite direction (i.e. randomly).

### Superoxide release assay

Superoxide anion levels were measured using an assay kit (from Sigma Aldrich) detecting chemiluminescence emanating from oxidation of luminol substrate by released superoxide anions. Freshly isolated neutrophils (10^6^ cells/100μl) suspended in assay medium were added to reaction mixtures containing assay buffer, luminol, enhancer and superoxide dismutase in triplicate wells of a 96 well plate. Additional wells were prepared with neutrophils alone (vehicle treated) and neutrophils stimulated with WKYMVm in the presence of superoxide dismutase (SOD). Assay buffer and assay medium alone served as a blank for the entire experiment. The reaction was initiated by addition of 10 μM WKYMvm as superoxide anion inducer. Luminescence intensity was measured immediately at 1 min intervals for 30 min using a Thermo Electron Corporation AppliSkan 2.3 Luminometer set at 37°C in high sensitivity mode.

### Statistical analysis

Data were analyzed using GraphPad Instat software (Calfornia, USA). Differences between groups were assessed using one-way ANOVA followed by Bonferroni post-hoc test, with p < 0.05 being regarded as significant.

## Results

### Effect of E121 treatment on colitis severity

**Fig 1:**
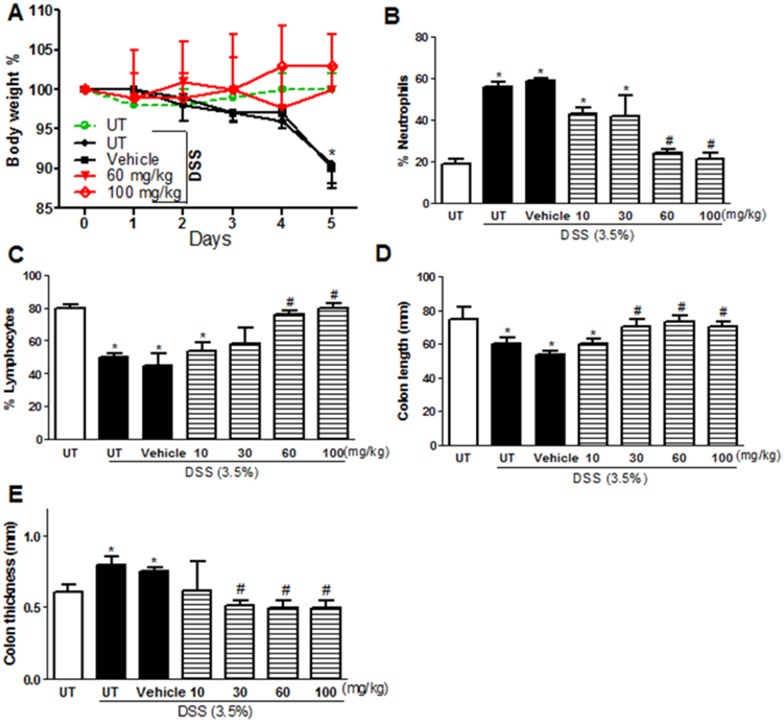
Effect of E121 treatment on colitis severity. Panel A shows % body weight changes in DSS treated mice alone or receiving vehicle (solid circles and squares, black lines), or E121 treated mice (red lines) compared to untreated (UT) mice receiving tap water only (open circle, hatched green line). Panels B and C show % of circulating neutrophils and lymphocytes respectively determined at day 5 post colitis induction in DSS treated mice alone or receiving vehicle (solid bars) or E121 treated mice (hatched bars) compared to UT group (open bars). Colon length (panel D) and thickness (panel E) was determined in all groups. Histobars represent means ± SEM for 6 mice in each group. Asterisks denote significant difference from UT mice with p<0.05 (*), # denotes significant difference from DSS alone and DSS/i.p vehicle treated mice with p<0.05.

The effect of E121 on modulating colitis severity in mice was tested by daily i.p injections of various doses of E121 (10–100 mg/kg). DSS administration alone or along with vehicle i.p (for 5 days) resulted in a significant drop in body weight (in contrast with mice receiving tap water only; UT). E121 at doses of 10 and 30 mg/kg has a similar drop in body weight compared to vehicle treated mice (data not shown). However, E121 treatment at doses of 60 and 100 mg/kg recovered the drop in body weight to similar levels seen in UT healthy mice (Fig 1A). DSS treatment alone or in vehicle i.p group resulted in increased circulating neutrophils and decreased circulating lymphocytes compared to UT mice; this was prevented by E121 treatment at doses of 60 and 100 mg/kg (Fig 1B and 1C). The decrease in colon length (Fig 1D) and the increase in colon thickness (Fig 1E) seen after DSS treatment was prevented by E121 treatment at doses of 30–100 mg/kg.

As indicated in Table 1, at the gross (macroscopic) level, DSS administration for 5 days alone or with daily i.p injections of vehicle resulted in diarrhea, blood in stool, erythema, edema and ano-rectal bleeding, none of which was seen in the UT group. Daily administration of E121 at doses of 30–100 mg/kg significantly reduced all of these effects except edema formation.

**Fig 2:**
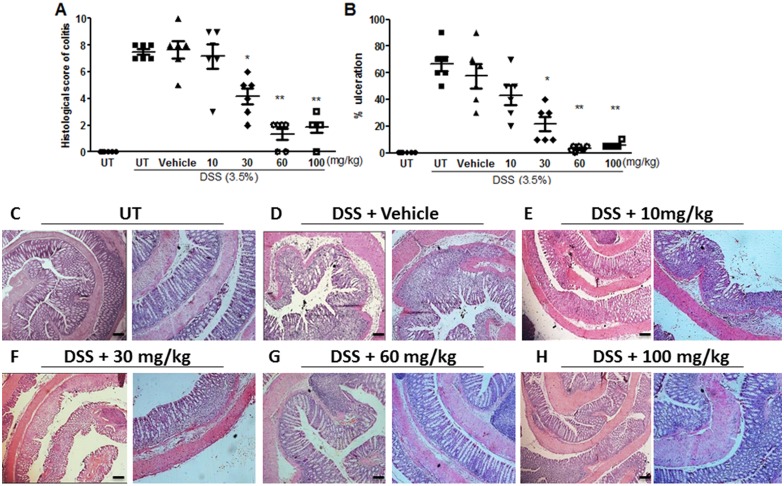
Effect of E121 treatment on the histological score for colitis severity. Panels A and B represent histological assessment of colitis severity and the % of ulceration in the whole colon section respectively in the groups indicated. Histobars represent means ± SEM for 6 mice in each group. Asterisks denote significant difference from UT mice with p<0.05 (*) and, p<0.001 (**). Panel C illustrates a colon section taken from UT mouse showing normal regular mucosal architecture. Panel D illustrates a colon section taken from mouse treated by daily i.p vehicle plus DSS where there is significant mucosal destruction and immune cell recruitment. Panels E-H illustrate typical colon sections taken from mice treated with various doses of E121 plus DSS. Treatment with 60–100 mg/kg doses of E121 improved mucosal integrity and reduced the enhanced immune cell recruitment (panels G and H). Bars represent 150 μm. Left pictures were taken at 5x magnification, while right pictures were taken at 10x magnification.

The histological (microscopic) score of colitis severity correlated well with the macroscopic score (as shown in Fig 2A); a significant reduction in colitis severity was seen with E121 treatment at doses of 30–100 mg/kg. Similarly, the % of ulceration (Fig 2B) was significantly reduced with E121 treatment at the same dose range. The histological features of the resected tissues are illustrated in Fig 2C–2H. DSS/i.p vehicle treatment for 5 days resulted in significant mucosal destruction and infiltration by immune cells (Fig 2D). Daily i.p injection of DSS treated animals with E121 at doses of 10–30 mg/kg slightly improved mucosal integrity and reduced the degree of immune cell recruitment to the colon (Fig 2E and 2F). E121 treatment at doses of 60–100 mg/kg significantly improved mucosal integrity and reduced the degree of immune cell recruitment to the colon (Fig 2G and 2H) to similar levels seen in UT healthy mice (Fig 2C).

### Effect of E121 treatment on the circulating levels of various cytokines

**Fig 3:**
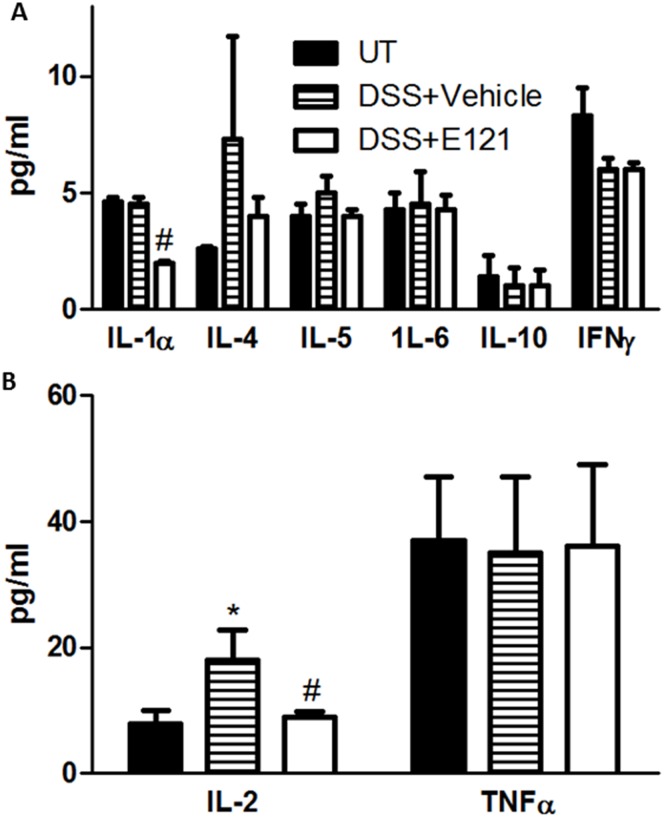
Effect of E121 treatment of circulating levels of various cytokines. Plasma levels (pg/ml) of various cytokines were measured in UT mice (solid bars), or mice treated with DSS plus vehicle (hatched bars) or E121 (60 mg/kg, open bars). Histobars represent means ± SEM for 4 mice in each group. Asterisk denotes significant difference from UT mice, with p<0.05, and # denotes significant difference from DSS/ i.p vehicle treated mice, with p<0.05.

There was no significant difference in the circulating levels of IL-5, -6, -10, and TNFα between the tested groups. A trend of reduced plasma level of IFNγ was observed in DSS treated compared to UT mice (Fig 3A). IL-1α was significantly reduced in DSS plus E121 treated mice compared to the other groups (Fig 3A). A trend of increased plasma levels of IL-4 in DSS treated mice and a trend of reduced IL-4 levels in DSS plus E121 treated mice compared to UT mice was observed (Fig 3A), which reached statistical significance for IL-2 (Fig 3B).

### Effect of E121 on expression/phosphorylation levels of molecules involved in signaling and apoptosis

**Fig 4:**
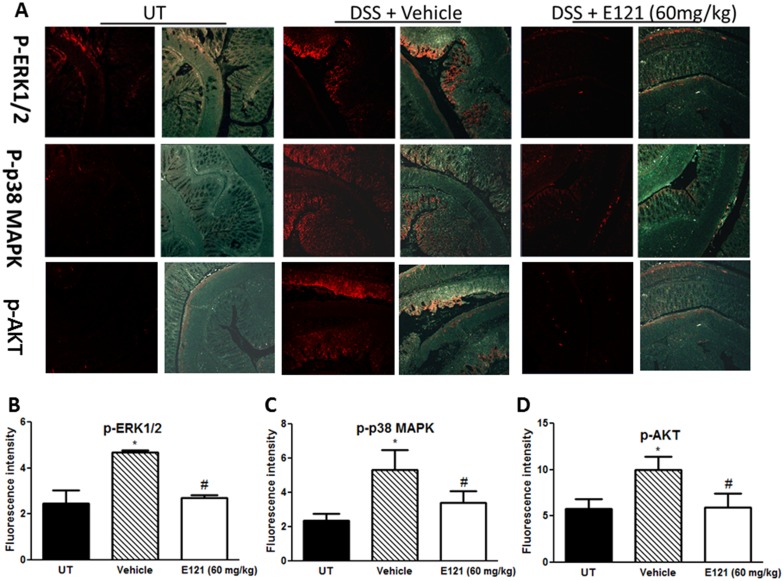
Effect of E121 on phosphorylation level of signaling molecules. Colon sections taken from untreated (UT) mice or mice treated with DSS for 5 days plus E121 (60 mg/kg) or vehicle were immunostained with antisera against phosphorylated ERK 1/2, p38 MAPK, and AKT (panel A). Immunofluorescent (Alexa Fluor) signals shown in left side of panel A are overlaid with DAPI stain on right side to show tissue architecture for the conditions indicated. Panels B-D represent quantitative assessment of fluorescence intensity (arbitrary units). Histobars represent means ± SEM for 4 mice in each group. Asterisk denotes significant difference from UT mice, with p<0.05, and # denotes significant difference from DSS/ i.p vehicle treated mice, with p<0.05.

The levels of phosphorylated forms of three key signaling intermediates involved in colitis pathogenesis; ERK1/2, p38 MAPK, and AKT were measured by immunofluorescence (IF) in sections from resected colon tissue of untreated (UT) mice or mice treated with DSS (for 5 days) plus daily E121 (60 mg/kg; which significantly improved colitis severity at gross and histological levels) or vehicle treatment. In each case, expression was enhanced by DSS and reduced by E121 back to the basal levels observed in the UT group (Fig 4).

**Fig 5:**
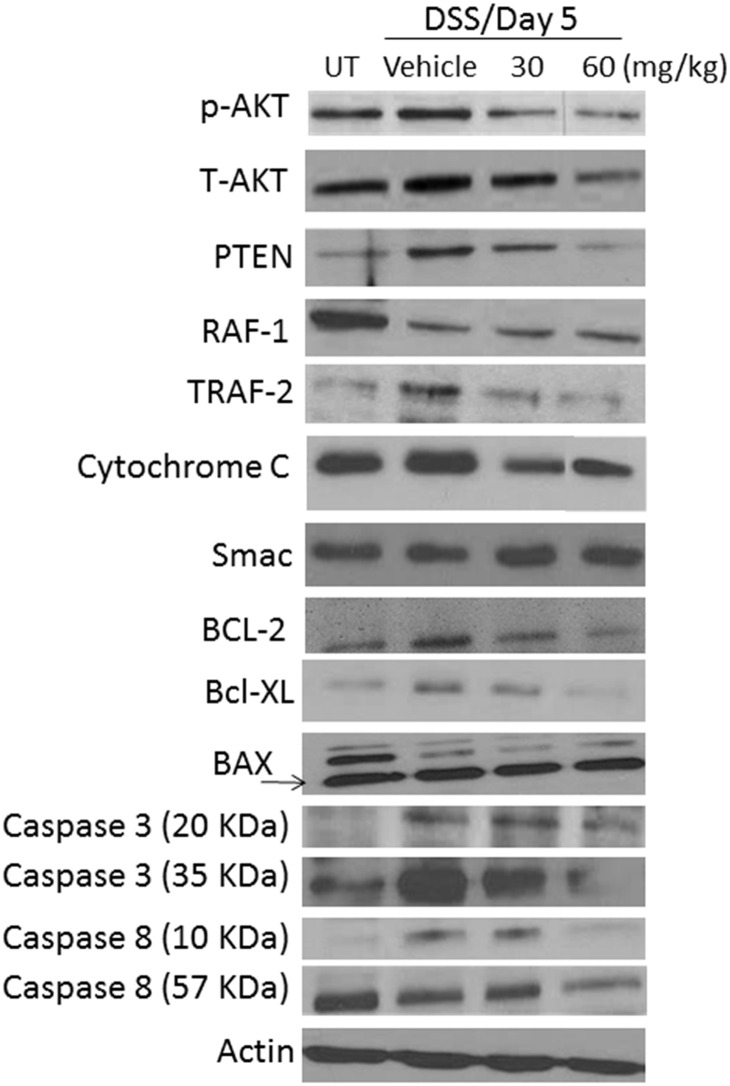
Effect of E121 on total/phosphorylated levels of proteins involved in cell signaling and apoptosis. Colonic levels of various proteins involved in cell signaling and apoptosis were determined by western blotting in UT or mice treated with either vehicle or E121 (30 and 60 mg/kg) at day 5 post colitis induction. The blots represent one of 3 similar experiments.

Western blotting analysis of colon homogenate (Fig 5) confirmed the modulated level of p-AKT seen in IF analysis (Fig 4A lower panel and Fig 4D). Enhanced expression levels of PTEN, total-AKT, TRAF-2, cytochrome C, BCL-2, BCL-XL, caspase 3 and caspase 8 were seen in DSS/vehicle i.p treated compared to UT mice, which was reduced with E121 treatment particularly at 60 mg/kg dose. The levels of RAF-1 were reduced in all treatment groups compared to UT mice. The levels of BAX and SMAC were not modulated in all of the tested groups. Actin bands indicate relatively equivalent amount of protein loaded in each condition.

### Effect of E121 treatment on the degree of neutrophil recruitment *in vivo*

**Fig 6:**
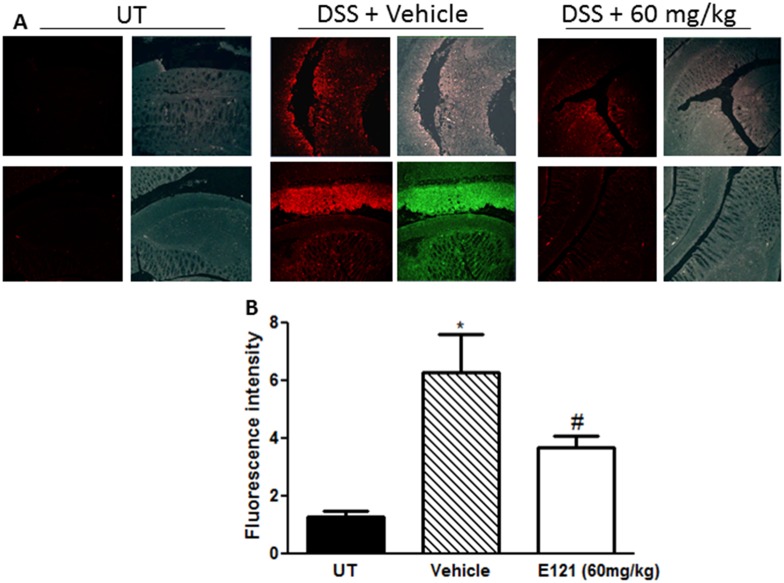
Effect of E121 on neutrophil recruitment *in vivo*. Colon sections taken from untreated (UT) mice or mice treated with DSS plus E121 (60 mg/kg) or vehicle were immunostained with antisera against the neutrophil marker Gr1 (panel A). Immunofluorescent (Alexa Fluor) signals shown in left side of panel A are overlaid with DAPI stain on right side to show tissue architecture for the conditions indicated. Panels B represent quantitative assessment of fluorescence intensity (arbitrary units). Histobars represent means ± SEM for 6 mice in each group. Asterisk denotes significant difference from UT mice, with p<0.05, and # denotes significant difference from DSS/ i.p vehicle treated mice, with p<0.05.

The degree of neutrophil recruitment to the colonic tissue was measured by IF (using the neutrophil marker Gr1 antibody) in sections from resected colon tissue of UT mice or mice treated with DSS (for 5 days) plus daily E121 (60 mg/kg) or vehicle treatment. Gr1 expression was significantly enhanced by DSS administration compared to UT group. E121 treatment (60 mg/kg) significantly reduced Gr1 colonic expression (Fig 6). These data demonstrate the integral role of neutrophils in colitis pathogenesis and the effect of E121 treatment on modulating the degree of cell recruitment to the colon.

### Effect of E121 on spontaneous neutrophil apoptosis *in vitro*

**Fig 7:**
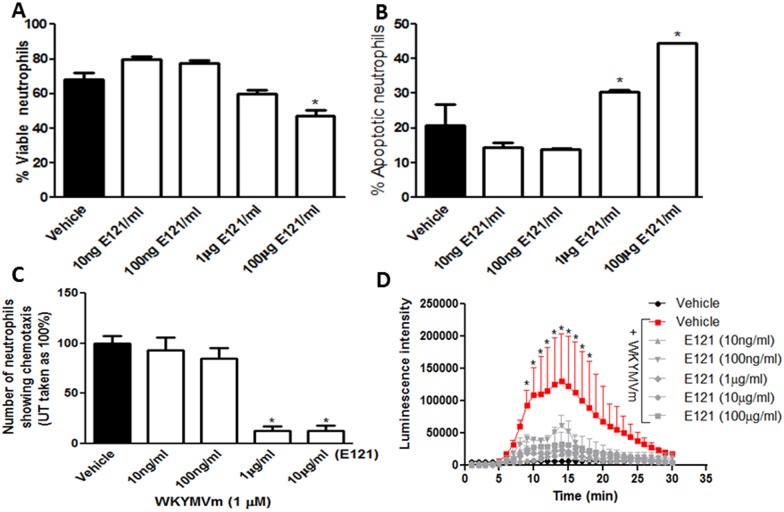
Effect of E121 on neutrophil apoptosis, chemotaxis, and superoxide release *in vitro*. Neutrophils were isolated from naïve mice and were treated with vehicle (solid bar) or various concentrations of E121 (open bars). Panels A and B represent % viable and % apoptotic cells. Panel C represents neutrophil chemotaxis, determined using the under agarose assay, towards WKYMVm (1μM) for vehicle treated (solid bar) or E121 treated (open bars) neutrophils. Panel D: superoxide released from neutrophils was determined by recording chemiluminescence emanating from oxidation of luminol substrate as described in Methods. Neutrophils were treated with vehicle only (solid circles), or 10 μM WKYMVm plus vehicle (solid squares) or various concentrations of E121 (gray lines). Histobars represent means ± SEMs for 3 independent experiments conducted with cells isolated from 3 mice in each group. Asterisks denote significant difference from vehicle group, with p<0.05.

E121 treatment significantly reduced the percentage of viable bone-marrow derived neutrophils (Fig 7A) and increased cell apoptosis (Fig 7B) relative to vehicle treated cells.

### Effect of E121 on neutrophil chemotaxis *in vitro*

E121 treatment (1–10 μg/ml) significantly decreased neutrophil chemotactic migration towards the bacterially-derived chemoattractant fMLP-like peptide (WKYMVm, 1 μM) as compared to vehicle treated neutrophils (Fig 7C).

### Effect of E121 on neutrophil superoxide release *in vitro*

As illustrated in Fig 7D, exposure of bone-marrow derived neutrophils to WKYMVm (10 μM) significantly enhanced superoxide release from neutrophils. This effect was abolished by co-treatment with superoxide dismutase (SOD, data not shown). Vehicle treated neutrophils did not induce superoxide release. Addition of E121 at doses of 10 ng/ml-100 μg/ml abolished WKYMVm-induced superoxide release.

## Discussion

In the present study, we showed for the first time experimental evidence suggesting anti-inflammatory properties for the enaminone E121 in the murine DSS colitis model. This effect was in part through modulation of plasma levels of IL-2, and colonic epithelial cell apoptosis and reduction in the activity of various pro-inflammatory signaling molecules such as ERK1/2, p38 MAPK and AKT. In addition, E121 significantly reduced *in vivo* neutrophil recruitment as well as *in vitro* neutrophil survival, and WKYMVm-induced neutrophil chemotaxis and superoxide release.

Apoptosis is considered a form of defensive mechanism against various inflammatory insults aiming to prevent excessive accumulation of non-functional cells in the tissue. This response is mediated through various molecules activated downstream either as intrinsic or extrinsic apoptotic pathways. Achieving a balance between the pro-apoptotic and anti-apoptotic molecules is required to maintain tissue homeostasis and will determine the cell fate. The intrinsic pathway is mediated through the release of cytochrome C from the mitochondria which interacts with Apaf-1 leading to the formation of apoptosome complex and the subsequent activation of caspases 9 and 3 [53–55]. Both the Bcl-2 family (pro-apoptotic proteins: Bax, Bad, Bak, Blk, Bid, Bim, and Noxa, and anti-apoptotic proteins: Bcl-2, Bcl-XL, and BAG) and the p53 tumor suppressor protein control the intrinsic apoptotic events [56]. The extrinsic pathway is mediated by signaling through the membrane bound death receptors which belong to the tumor necrosis factor (TNF) family [57]. Various domains are formed leading to caspase-8 activation, which either activate the intrinsic pathway through Bid or activate caspases 3 and 7 [58, 59]. Caspases are cysteine aspartic-specific proteases, which are classified into initiators (2, 8, 9, and 10), effectors (3, 6, 7, and 14), or cytokine activators (1, 4, 5, 11, 12, and 13) [60, 61]. Dysregulated apoptotic event has been linked with the pathogenesis of various conditions including cardiac ischemia-reperfusion injury [62], neurodegenerative diseases [63], and cancer [64]. In regards to IBD, an abundant presence of apoptotic colonic epithelial cells in active ulcerative colitis (UC) was documented in part due to increased nitric oxide synthesis [65–67] (which causes oxidative damage and DNA fragmentation) and over-expression of p53 [68, 69]. A report published by Iimura *et al* suggested no differences in Bcl-XL and BCL-2 protein expression, and downregulation in Bax expression in inflamed colonic mucosa of UC patients compared to un-inflamed regions or healthy controls [70]. Reduced Bax expression was suggested to be due to NFƙB-induced pro-inflammatory proteins (e.g. IL-6) which downregulate Bax gene expression in the inflamed mucosal surface of UC patients [71, 72]. The colonic levels of BCL-2 was shown to be reduced in the DSS [73–75], TNBS [76], and DNBS [77] models. However, other reports showed enhanced BCL-2 expression in the colonic epithelial cells of UC patients [78], and in mice post DSS [79] or TNBS [80] treatment. Using the TNBS model, mesalamine treatment reduced colitis severity in mice in part through reducing BCL-2 colonic expression levels [80], which is in agreement with our finding using the DSS model where E121 also reduced its expression in the colon homogenate (Fig 5). The colonic levels of BCL-XL was shown to be unchanged [81], reduced [76], or increased [82] in animal models of colitis. Furthermore, its expression was also increased in colon biopsies taken from IBD patients [83]. Our data are in agreement with enhanced BCL-XL colonic expression in active colitis (Fig 5). Cytochrome C level was shown to be modulated in animal models of colitis. Tirosh *et al* [84, 85] demonstrated reduced colonic cytochrome C levels at 24 h post TNBS administration in rats at both miRNA and protein levels, while Crespo *et al* [76] showed enhanced cytochrome C expression at 2–7 days post TNBS administration in rats, which was also shown in our study (Fig 5). The colonic levels of various caspases were shown to be increased post colitis induction. For example, the levels of caspase-3 [81, 82, 86], -7 [81] and -12 [76], and the cleaved form of caspases-1 [87, 88], -12, and -7 [73] were increased in the colonic tissues in various animal models of colitis, which are also in agreement with our findings in regard to caspase-3 and -8 levels (Fig 5).

The RAF proto-oncogene (c-RAF) is a serine/threonine-protein kinase [89] which is involved in cellular processes including proliferation, differentiation, and survival, and function as an activator of ERK 1/2 pathway. It is considered as an anti-apoptotic regulator in cardiomyocytes through the inhibition of the pro-apoptotic kinase apoptosis signal-regulating kinase-1 [90]. Enhanced phosphorylated levels of RAF was observed in the IL-10 deficient mice (which develop spontaneous colitis over time) during active disease [91]. Using the DSS colitis model, intestinal epithelium-specific RAF knockout mice developed significantly enhanced colitis severity compared to wild type mice due to increased levels of colon epithelial apoptosis, decreased enterocyte proliferation, and attenuated NF-κB activation following DSS treatment [92]. These results suggest the importance of RAF in regulating NF-κB activation and cell survival to protect against intestinal epithelial injury and inflammation [92]. We demonstrated reduced colonic levels of RAF-1 in all treatment groups compared to UT controls (Fig 5).

The tumor necrosis factor receptor-associated factor-2 (TRAF-2) act as an adaptor that transmit signals through the TNF receptor superfamily, IL-1, and Toll-like receptors [93, 94] resulting in NFƙB activation (which is required to prevent epithelial cell apoptosis). A recent report demonstrated that TRAF-2 deficient mice spontaneously developed severe colitis due to enhanced TNFα-induced apoptosis of colonic epithelial cells [95]. Treating these mice with neutralizing antibodies against TNFα and IL-10 ameliorated colitis severity and prolonged survival [96]. Furthermore, a recent study performed on IBD patients showed enhanced expression levels of TRAF-1 and -2 in the colonic mucosal of IBD patients compared to healthy controls. Moreover, inflamed tissues had higher TRAF-1 and -2 expression than non-inflamed tissues, suggesting that their altered expression level might be an early event in the disease pathogenesis [97]. We demonstrated enhanced TRAF-2 expression in the colon of DSS treated mice, and E121 treatment reduced its expression back to control level (Fig 5).

There is solid evidence regarding the importance of PI3K/MAPK signaling pathway in colitis pathogenesis. Enhanced AKT phosphorylation was evident in colonic biopsy of IBD patients and animal models of colitis, and treatment with wortmannin and AS605240 (PI3K inhibitors) ameliorated colitis severity [98, 99]. A recent report showed reduced colitis severity by treatment with the macrolide 7-O-succinyl macrolactin A (SMA), which was mediated in part through inhibiting TNF-induced PI3K, AKT, mTOR and p70S6 kinase phosphorylation [100]. Furthermore, the anti-inflammatory effects of the immunomodulating tellurium compound ammonium trichloro (dioxoethylene-o,o') tellurate (AS101) on the murine DSS model was dependent on PI3K/AKT signaling pathway [75]. We showed enhanced total and phosphorylated form of AKT in the colon of DSS treated mice, which was reduced to control levels by E121 treatment (Figs 5 and 4A and 4D). The reduced expression level of AKT in response to E121 treatment may also be due to reduced immune cell requirements to the colonic tissues (e.g. neutrophils as shown in Fig 6) and not solely dependent on reduced colonic epithelial cell apoptosis. Furthermore, all of the three MAPK family; p38 MAPK, JNK, and ERK1/2 have been suggested to play a role in IBD pathogenesis. Their enhanced activity was demonstrated in colonic biopsy taken from IBD patients, and treatment with inhibitors for MAPK pathway ameliorated pro-inflammatory cytokine levels (e.g. TNFα) and disease activity index leading to endoscopic improvement [101, 102]. A recent report showed that the anti-inflammatory and anti-oxidant effects of paeonol, and its metabolites was mediated through reduction of p-ERK/p38 MAPK levels in macrophages and consequently reducing pro-inflammatory cytokine release (e.g. TNFα) [102]. Various pro-inflammatory cytokines like TNFα also induce intestinal epithelial cell (IEC) apoptosis contributing to colonic tissue injury after TNBS administration in mice. It has been shown that the TNF- induced IEC apoptosis was ameliorated by treatment with the serum-and-glucocorticoid-inducible-kinase-1 (SGK1) in part through triggering MEK/ERK activation [103]. Interestingly, the mucosal repair properties of insulin-like growth factor-1 (IGF-1) after colitis induction by DSS administration in mice was shown to be through β-arrestin2-mediated ERK signaling [104]. We showed enhanced colonic expression levels of p-ERK1/2 and p-p38 MAPK post DSS treatment which was reduced to control levels by E121 treatment (Fig 4).

The evidence for the role of oxidative stress in IBD pathogenesis is also strong. Enhanced colonic tissue levels of myeloperoxidase activity (a neutrophil-rich enzyme) has been reported in various animal models of colitis as well as in IBD patients [105, 106]. This is due to enhanced neutrophil recruitment and/or delayed apoptosis at the inflammatory site contributing to the disease pathogenesis [30]. *Ex vivo* apoptosis was significantly delayed in neutrophils isolated after the induction of colitis using an oligofructose-overdose model, which was evident by the reduction in caspase-3, -8, and -9 activities [107]. Furthermore, depleting circulating neutrophils (by neutralizing antibody treatment) [108, 109], or the administration of antioxidants (such as superoxide dismutase) significantly reduced colitis severity [105], suggesting the importance of neutrophils and their reactive metabolites in IBD pathogenesis. E121 treatment significantly reduced neutrophil spontaneous apoptosis (Fig 7A and 7B), and WKYMVm-induced neutrophil chemotaxis (Fig 7C) and superoxide release (Fig 7D), suggesting that its anti-inflammatory properties are in part through modulation of neutrophil effector functions.

In conclusion, E121 might serve a potential therapeutic target for IBD treatment. Its anti-inflammatory property is in part mediated through modulating the activity of pro-inflammatory signaling pathways, colonic epithelial cell apoptosis, and neutrophil apoptosis, chemotaxis, and superoxide release.
